# Time course of costamere-related alterations in focal adhesion signaling and composition of rat soleus muscle after achilles tenotomy

**DOI:** 10.1016/j.dib.2019.103999

**Published:** 2019-07-10

**Authors:** Céline Ferrié, Stephanie Kasper, Florian Wanivenhaus, Martin Flück

**Affiliations:** a Laboratory for Muscle Plasticity, Department of Orthopedics, University of Zurich, Balgrist Campus, Zurich, Switzerland; b Department of Orthopedic Surgery, Balgrist University Hospital, Zurich, Switzerland

## Abstract

Sarcolemma-based focal adhesions (costameres) are a central hub for the cytoskeletal anchoring of myofibrils and mechano-regulated signaling. Here we report the time course of alterations in focal adhesion-associated signaling and fiber composition in rat soleus muscle after Achilles tenotomy. The report includes data from tenotomized muscles and contralateral mock controls to expose whether muscle degeneration after tenotomy is due to the transection of the Achilles tendon, or circumjacent surgical manipulations of the tendon. With respect to the interpretation of the data regarding mechanistic implications of costamere-associated processes for surgical repair of the detached muscle-tendon complex the reader is referred to the accompanying research article ‘Focal adhesion kinase coordinates costamere-related JNK signaling with muscle fiber transformation after Achilles tenotomy and tendon reconstruction’ Ferrié et al., 2019.

**Table:** Specifications table

Subject area	*Biology*
More specific subject area	*Muscle physio-pathology, biochemistry*
Type of data	*Figure with Box Whisker plots, biochemical and histochemical data.*
How data was acquired	*Electrochemiluminescence-based immunoassay (QuickPlex SQ120 Imager, Meso Scale Discovery, USA)* *SDS-PAGE immunoblotting (Pxi, Syngene USA)* *Microscope (IX50 microscope, Olympus, USA)*
Data format	*Recorded tiff-files, analyzed as Box Whisker plots*
Experimental factors	Tenotomy versus mock-tenotomy, multiple time points, positive and negative control for gene plasmids
Experimental features	*The intervention comprised a time course experiment to measure the effect of unilateral Achilles tenotomy on soleus muscle composition compared to the mock-treated muscle from the contralateral leg in rats. Animals were allowed to recover for 5 minutes, 4 days or 14 days after tenotomy before the soleus muscle pairs were collected under anesthesia. Cryosections were prepared from the belly portion of the muscles and subjected to histological analysis of muscle composition, and pooled to prepare homogenate that was used in the biochemical analysis of protein expression by immunoblotting and electrochemiluminescence-based immunoassay*.
Data source location	*Zurich, Switzerland, 47°21′10″ N 8°34′28″ O*
Data accessibility	*Examples of recorded immunoblotting data and graphical representations of the data distribution are shown. The raw data set is publicly available under*https://doi.org/10.17632/hdmgydpr3v.1*after expiration of the embargo period (14-05-2019).*
Related research article	*Ferrié, C., S. Kasper, F. Wanivenhaus, M. Flück (2019). “Focal adhesion kinase coordinates costamere-related JNK signaling with muscle fiber transformation after Achilles tenotomy and tendon reconstruction.” Experimental and Molecular Pathology 108: 42–56.*

**Table:** 

**Value of the data** • The data show the temporal course of alterations in muscle fiber composition and focal adhesion signaling subsequent to tendon release. • The data expose molecular and cellular effects that are induced in anti-gravity muscle with the surgical manipulation of the Achilles tendon. • The data identify, and rule out, certain molecular and cellular parameters as hallmarks of tenotomy-induced muscle degeneration. • The data are useful to judge the window of opportunity for surgical repair of torn tendons to minimize the deterioration of detached anti-gravity muscle.

## 1 Data

**Fig. 1:**
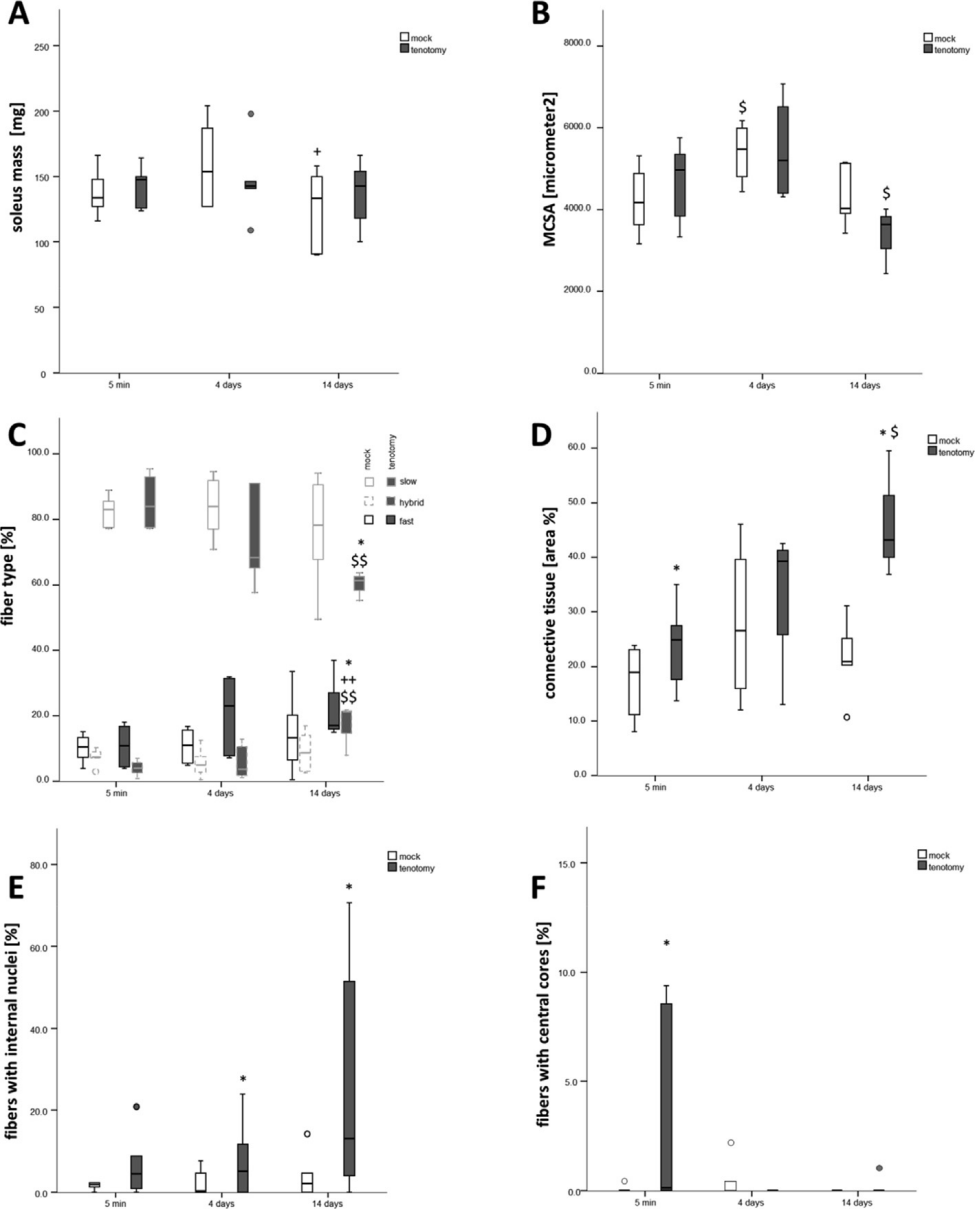
Time course of effects of Achilles tenotomy on the anatomy of soleus muscle. Box Whisker plots of anatomical parameters of *soleus* muscles, mass (A), MCSA of muscle fibers (B), fiber type percentage (C), connective tissue area percentage (D), percentage of fibers with internal nuclei (D) and fibers with central cores (F), 5 minutes, 4 days or 14 days after tenotomy, and the respective mock controls. Circles indicate outliers. * and **, p < 0.05 and p < 0.005 vs. mock, $ and $$, p < 0.05 and p < 0.005 vs. 0 min, + and ++, p < 0.05 and p < 0.005 vs. 4 days (repeated-measures ANOVA).

**Fig. 2:**
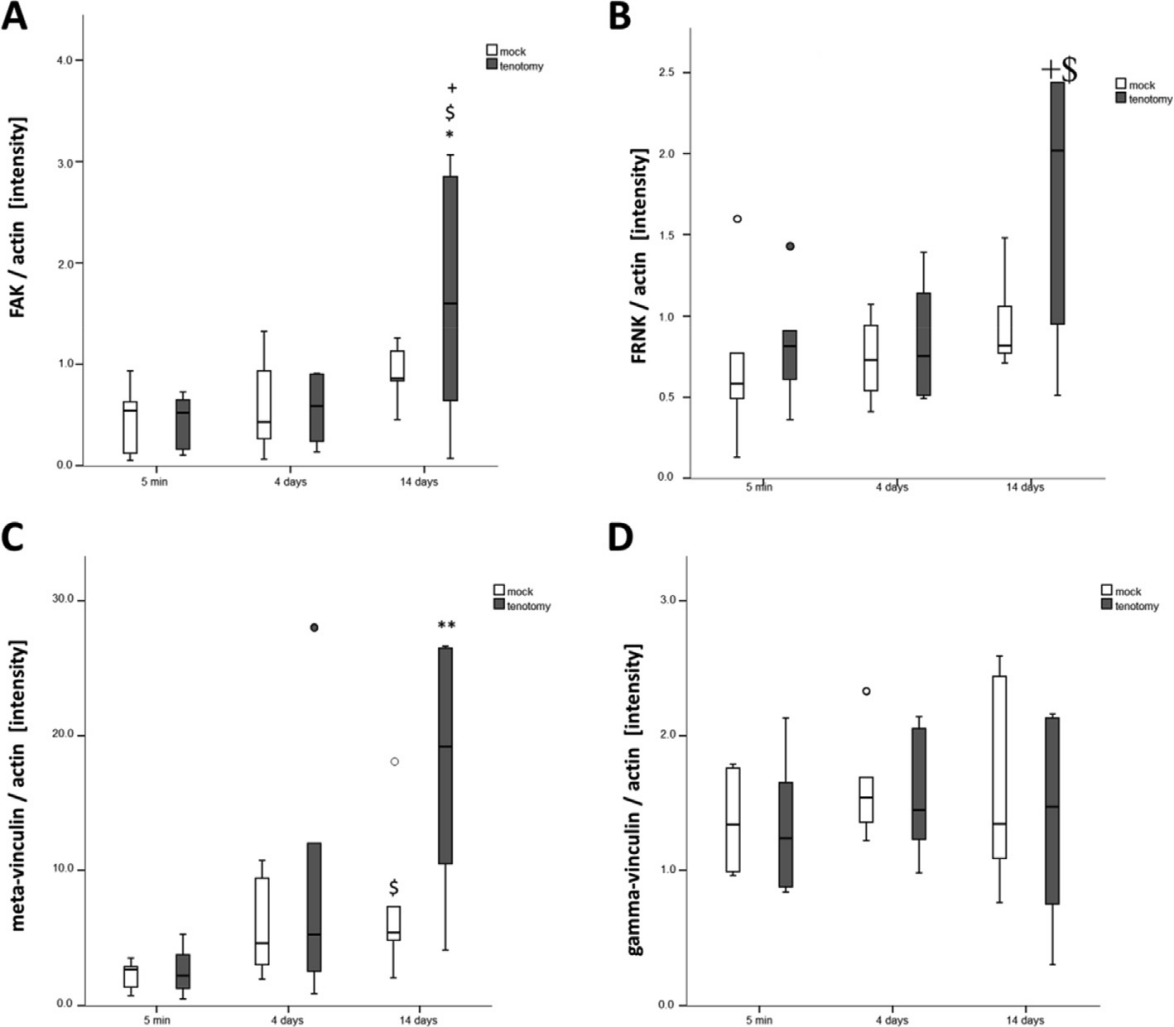
Time course effects of tenotomy on costamere component expression in soleus muscle. Box Whisker plots of expression levels for costameric proteins FAK (A), FRNK (B), meta-vinculin (C), and gamma-vinculin (D), in *soleus* muscles 5 minutes, 4 days or 14 days after tenotomy, and the respective mock controls. Circles indicate outliers. * and **, p < 0.05 and p < 0.005 vs. mock, $ and $$, p < 0.05 and p < 0.005 vs. 0 min, + and ++, p < 0.05 and p < 0.005 vs. 4 days (repeated-measures ANOVA). See Fig. 4 for representative immunoblots.

**Fig. 3:**
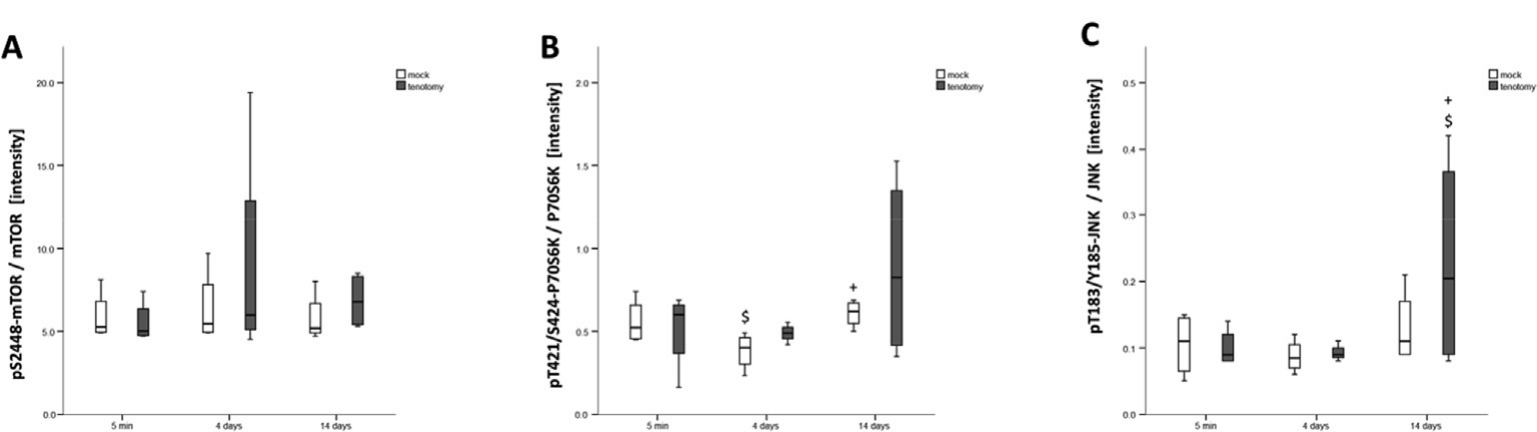
Time course effects of tenotomy on hypertrophy and immediate early gene signalling in soleus muscle. Box Whisker plots of the specific phosphorylation of the mTOR (A), P70S6K (B) and JNK (C) in *soleus* muscles 5 minutes, 4 days or 14 days after tenotomy, and the respective mock controls. Circles indicate outliers, respectively. * and **, p < 0.05 and p < 0.005 vs. mock, $ and $$, p < 0.05 and p < 0.005 vs. 0 min, + and ++, p < 0.05 and p < 0.005 vs. 4 days (repeated-measures ANOVA).

Graphical representations of the distribution of the values as measured 5 minutes, 4 days and 14 days after Achilles tendon release in tenotomized and mock-tenotomized rat soleus muscle are shown for anatomical parameters of muscle composition (mass, MCSA of muscle fibers, fiber type percentage, connective tissue area percentage, percentage of fibers with internal nuclei and fibers with central cores; Fig. 1), protein levels of costamere-associated factors (Fig. 2), and specific phosphorylation levels of T183/Y185-JNK, T421/S424-P70S6K and S2448-mTOR (Fig. 3).

**Fig. 4:**
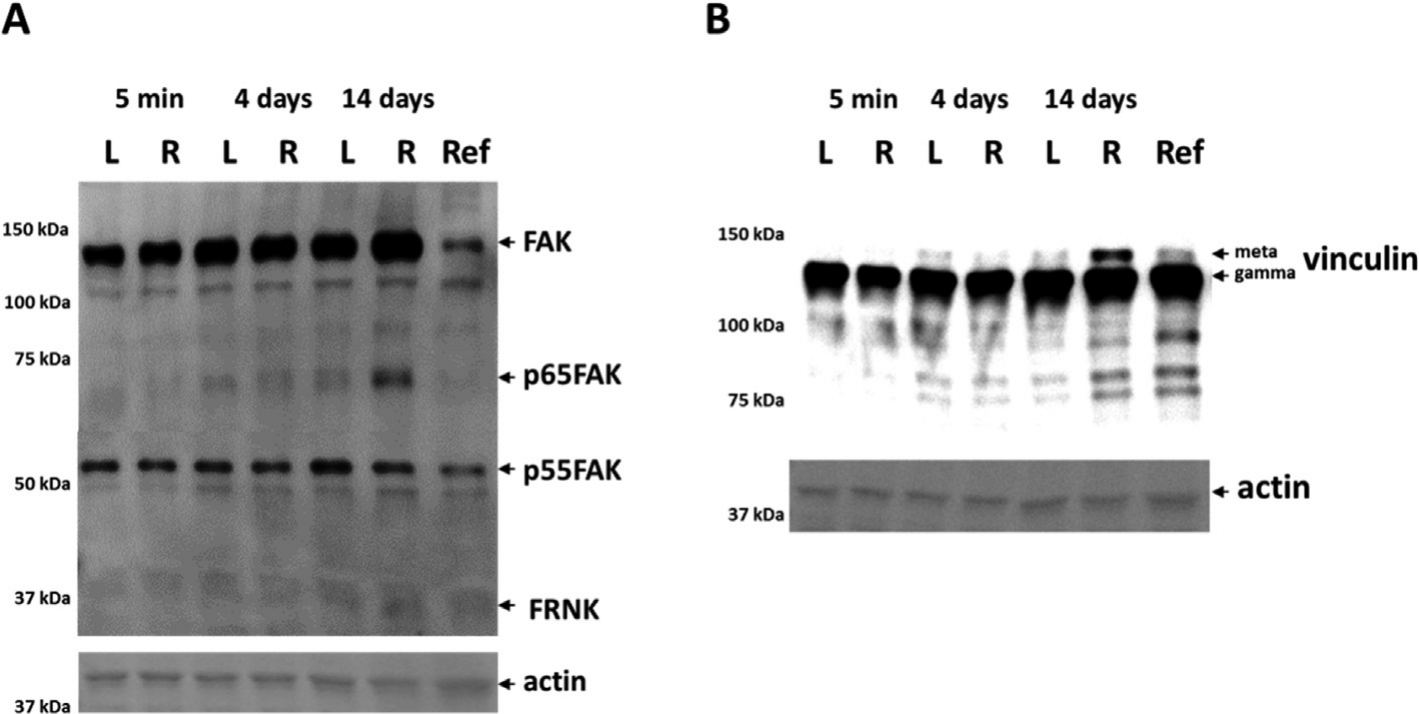
*Costamere component expression in rat soleus muscle after tenotomy.* Example western blots showing the detection of FAK and FAK C-terminus related proteins (A) and vinculin isoforms (B) in one sample each from a tenotomized soleus muscle (R) and its mock control (L) after different duration after Achilles tenotomy. Below, actin staining of the respective blot. Ref: reference sample. Note the low abundant levels of FRNK.

The data show tenotomy-specific, and in part transient reactions on anatomical parameters (the distribution of fiber types, fibers with internal nuclei, connective tissue; Fig. 1), levels of costamere-associated cytoskeletal proteins (FAK, meta-vinculin; Fig. 2) and the phosphorylation of focal adhesion-associated signalling proteins (T183/Y185-JNK, T421/S424-p70S6K; Fig. 3). Reactions in mock controls were seen for MSCA of muscle fibers 4 days after tenotomy. The percentage of fibers with central cores was rapidly, but not robustly, increased in tenotomized muscle (Fig. 1F). Fig. 4 depicts examples for the detection of costamere component expression in rat soleus muscle after tenotomy.

## 2 Experimental design, materials and methods

### 2.1 Experimental design

The experiment comprised measures on the temporal effect of unilateral Achilles tenotomy on rat *soleus* muscle 5 minutes, 4 days and 14 days after tenotomy [1]. These sample points were chosen because the same durations reportedly produced effects on FAK-associated signaling molecules in rat *soleus* muscle with Achilles tenotomy and muscle unloading or manifest in structural degeneration of *soleus* muscle [2], [3], [4], [5], [6], [7]. Possible compensatory effects in agonistic muscle groups (reviewed in [8]) were controlled by comparing assessed parameters in the tenotomized right *soleus* muscle to mock manipulations in the contralateral controls. The experiment was performed in 3 series, whereby the animals were randomly assigned to groups of six animals, which were subjected to tenotomy and thereafter allowed to recover for a same given duration, i.e. 5 minutes, 4 days or 14 days before the *soleus* muscles were collected under anesthesia from the tenotomized and mock-treated leg. For the 4 day and 14 day time point, the wound was closed, and animals allowed moving freely before muscles were collected. Cryosections were prepared from the belly portion of the collected muscles, and subjected to histological analysis of muscle composition, and pooled for biochemical analysis of protein expression by immunoblotting and electrochemiluminescence-based immunoassay from muscle homogenate.

### 2.2 Animal care

The experimental procedures were approved by the animal protection commission and ethics commission of the Canton of Zurich, Switzerland and conducted in facilities being approved by the Institutional Animal Care and Use Committee. Twelve-week old Wistar female rats (Janvier Labs, France) were housed in a temperature and humidity-controlled facility. Upon arrival and before the surgical procedure (start), animals were acclimatized for 1 week in new cages in groups of three animals in a cage. After tenotomy animals were housed as pairs. The healthiness and the stress level of each rat were monitored daily over the entire duration of the experiment with a score table.

To reduce pain, doses of analgesic (Buprenorphin 50 μg/kg body weight (Temgesic, Reckitt Benckiser AG, Switzerland)) were administered subcutaneously half an hour before the operation took place and 5 min before inhalation anesthesia was terminated. Further doses of analgesic were administered during a 24h-follow-up according to the postoperative pain monitoring.

### 2.3 Tenotomy

These experiments were performed essentially as described before [1], [6]. Rats were anesthetized with 2–4% isoflurane (Rothacher-Medical GmbH, Switzerland, #ISO250ml) and oxygen (1L min-1). Anesthesia was maintained in 3% isoflurane during the entire surgical procedure with the help of a commercial tube system (Provet, Lyssach b. Burgdorf, Switzerland).

On the right leg of each animal, an approximately 4-mm-long incision was made at the dorsal side of the lower leg towards the tendinous end of the *gastrocnemius* muscle taking care underlying blood vessels and nerves were not damaged. The Achilles tendon was exposed by lifting the *soleus* portion of the Achilles tendon with a pair of forceps and the tendon was transected with a scalpel. Subsequently the skin is closed with a Novosyn^®^ thread (5-0, DS16, Braun Medical AG, Switzerland). Left muscles did undergo a mock tenotomy in which the *soleus* muscle tendon was exposed, but not dissected. Subsequently, animals were released from the anesthesia and allowed to gain consciousness under normal oxygenation in a cage.

### 2.4 Muscle collection

At the end of the experiment, rats were anesthetized in 2–4% isoflurane and a circular incision was drawn around the heel just above the calcaneus followed by a long medial cut from the heel towards the knee. Subsequently, the musculature of the lower leg was exposed by stripping the skin towards the knee. The two tendons that insert distally into the lateral and medial head of the *gastrocnemius* muscle were clamped and then the *soleus* muscle was exposed by reverting the triceps group over the popliteal fossa. The *soleus* muscle was grabbed with a pair of forceps and its proximal tendinous end was capped with a scalpel. The collected muscle was weighed in a microbalance before being frozen in liquid nitrogen-cooled isopentane (VWR International GmbH, Switzerland, #24872.298). The same procedure was carried out for the left *m. soleus*. All muscles were kept in sealed cryo-tube at -80 °C freezer until molecular analysis was performed.

### 2.5 Muscle sectioning

Cryosections were prepared in perpendicular direction to the major axes of *soleus* muscles with the help of a cryostat (CM3050, Leica, Germany). The ten first sections were laid on slides (Menzel, Superfrost) for future staining. The following one hundred sections were collected into two separate flat bottom tubes (Eppendorf, Faust Laborbedarf AG, 8200 Schaffhausen, Switzerland), which were cooled in liquid nitrogen, and stored until further biochemical analysis (i.e. immunoblotting) at -80 °C.

### 2.6 Histological analysis of muscle fiber type composition and cross-sectional area

To quantify fast and slow fibers, two continuous sections of the right and left *soleus* were stained of each rat with antibodies against slow (Merck Millipore Corporation, Switzerland, #MAB1628) and fast type myosin heavy chain (Sigma-Aldrich Chemie GmbH, Switzerland, #M4276), respectively. Then, overlapping images were taken at a 10X magnification (Olympus IX50 microscope, USA) from the stained sections and assembled to reveal full coverage of the section. Image J software (version 1.46, National Institutes of Health, USA) was used to determine the number of fast, slow or hybrid fibers in each section. Cryosections of right and left *soleus* were processed using the Goldner trichrome method. Overlapping images were taken at a 10X magnification (Olympus IX50 microscope, USA), assembled and analyzed based on morphometric principles [9]. In brief, a grid was superimposed and the images printed at a final 7700-fold magnification on DIN A3 paper. The number of muscle fiber types, and fibers with internal nuclei or central cores was estimated in every fourth square under application of the forbidden line rule. Subsequently the squares were subdivided into 16 equal sized squares and the mean cross-sectional area (MCSA) of fibers was estimated by point counting of the fiber profiles. On average 299 fibers were evaluated per *soleus* muscle.

### 2.7 Homogenate preparation

Frozen sections were homogenized in 200 μL of RIPA buffer (2% triton 100X, 1% NP-40, 300mM NaCl, 20mM Tris base, 2mM EDTA, 2mM EGTA) with a Polytron^®^ (PT 1200 E, Kinematica AG, Switzerland). Total protein content was determined with the Pierce^®^ BCA protein assay kit (Thermo Fischer Scientific, USA).

### 2.8 Immunoblotting

10 μg protein of each homogenates, was analyzed for the content of FAK, FRNK, gamma- and meta-vinculin by SDS-PAGE, subsequent immunoblotting and enhanced chemoluninescence-based signal detection essentially as described using established antibodies [6], [10], [11]. The dilutions of the first antibodies were 1:1000 for the polyconal antiserum LuLu to detect FAK and FRNK [12], as well as the polyclonal antibody against vinculin to gamma- and meta-vinculin (Sigma-Aldrich Chemie GmbH, Switzerland, #V9264). The dilutions for the appropriate horseradish peroxidase-conjugated goat secondary antibodies against rabbit IgG (#55676, MP Biomedicals, Zurich, Switzerland) or against mouse IgG (#9917, Sigma, Buchs, Switzerland) were both 1:10000. Back-ground corrected band intensities were related to the intensity of the actin band for the respective homogenate sample as identified on the Ponceau S stained membrane as described. Samples were loaded with a specific analytic scheme comprising the inclusion of a reference sample on each blot. For each immunoblot the actin-related values were related to the respective values from the reference samples and the resulting values from the different immunoblots were pooled.

### 2.9 Electrochemiluminescence-based immunoassay

Multiplex kits using electro-chemiluminescence technology were used to quantify the phosphorylation of selected signaling factors through the measure of critical auto-regulatory sites. This comprised P70S6K (T421, S424), mTOR (S2448), JNK (T183, Y185), and respective total proteins (K15114D-1, K15170D-1 and K15111D-1, Meso Scale Discovery, USA) in supernatants of RIPA-based homogenates of *soleus* muscle. Plates were read on a QuickPlex SQ120 Imager (Meso Scale Discovery, USA) as per the manufacturer's instructions. Homogenate corresponding to 33 μg of protein was loaded per well of the ELISA plate. The results of the ELISA were expressed in ECL counts and the values of phospho-protein were related to respective total protein expression of each kinase, that is, JNK, mTOR and P70S6K to reveal the specific phosphorylation levels. As this was effectively a ratio of ECL counts, no units were assigned to phospho-protein levels. The P70S6K assay was validated against reported measures from immunoblotting signals in muscle samples [13].

### 2.10 Statistical analysis

Data were organized using MS-Excel (Microsoft, Kildare, Ireland) and exported into SPSS (version 23, IBM) to carry out statistical tests. A repeated measures ANOVA for the repeated factor leg (mock, tenotomy) was performed to test the effects of tenotomy on the expression levels of the individual proteins and respective levels of phosphorylation. A repeated ANOVA for the repeated factor leg (mock, tenotomy) and the factor time (5 min, 4 days, 14 days) was performed to test the interaction effects of time x tenotomy. Subsequently, effects demonstrating a p-value below 0.05 were localized post-hoc with a test for least significance difference. Data were displayed as median ± standard deviation and minima/maxima in Box Whisker plots.

## References

[1] Ferrié C., Kasper S., Wanivenhaus F., Flück M. (2019). Focal adhesion kinase coordinates costamere-related JNK signaling with muscle fiber transformation after Achilles tenotomy and tendon reconstruction. Exp. Mol. Pathol..

[2] Aronson D., Dufresne S.D. (1997). Contractile activity stimulates the c-Jun NH2-terminal kinase pathway in rat skeletal muscle. J. Biol. Chem..

[3] Baker J.H., Hall-Craggs E.C. (1980). Recovery from central core degeneration of the tenotomized rat soleus muscle. Muscle Nerve.

[4] Jakubiec-Puka A., Catani C. (1992). Myosin heavy-chain composition in striated muscle after tenotomy. Biochem. J..

[5] Jamali A.A., Afshar P. (2000). Skeletal muscle response to tenotomy. Muscle Nerve.

[6] Klossner S., Li R. (2013). Quantitative changes in focal adhesion kinase and its inhibitor, FRNK, drive load-dependent expression of costamere components. Am. J. Physiol. Regul. Integr. Comp. Physiol..

[7] Midwood K.S., Schwarzbauer J.E. (2002). Tenascin-C modulates matrix contraction via focal adhesion kinase- and Rho-mediated signaling pathways. Mol. Biol. Cell.

[8] Lu D.X., Kaser L. (1999). Experimental changes to limb muscles elicit contralateral reactions: the problem of controls. J. Exp. Biol..

[9] Mayhew T.M. (1991). The new stereological methods for interpreting functional morphology from slices of cells and organs. Exp. Physiol..

[10] Franchi M.V., Ruoss S. (2018). Regional regulation of focal adhesion kinase after concentric and eccentric loading is related to remodeling of human skeletal muscle. Acta Physiol..

[11] Ruoss S., Mohl C.B. (2018). Costamere protein expression and tissue composition of rotator cuff muscle after tendon release in sheep. J. Orthop. Res..

[12] Fluck M., Carson J.A. (1999). Focal adhesion proteins FAK and paxillin increase in hypertrophied skeletal muscle. Am. J. Physiol..

[13] Klossner S., Durieux A.C. (2009). Mechano-transduction to muscle protein synthesis is modulated by FAK. Eur. J. Appl. Physiol..

